# Effect of Enterotoxigenic *Escherichia coli* on Microbial Communities during Kimchi Fermentation

**DOI:** 10.4014/jmb.2108.08038

**Published:** 2021-09-08

**Authors:** Woojung Lee, Hyo Ju Choi, Hyunwoo Zin, Eiseul Kim, Seung-Min Yang, Jinhee Hwang, Hyo-Sun Kwak, Soon Han Kim, Hae-Yeong Kim

**Affiliations:** 1 Division of Food Microbiology, National Institute of Food and Drug Safety Evaluation, Ministry of Food and Drug Safety, Cheongju 28159, Republic of Korea; 2 Institute of Life Sciences and Resources and Department of Food Science and Biotechnology, Kyung Hee University, Yongin 17104, Republic of Korea

**Keywords:** Enterotoxigenic *E. coli* (ETEC), 16S rRNA gene, Kimchi, Microbial community

## Abstract

The diverse microbial communities in kimchi are dependent on fermentation period and temperature. Here, we investigated the effect of enterotoxigenic *Escherichia coli* (ETEC) during the fermentation of kimchi at two temperatures using high-throughput sequencing. There were no differences in pH between the control group, samples not inoculated with ETEC, and the ETEC group, samples inoculated with ETEC MFDS 1009477. The pH of the two groups, which were fermented at 10 and 25°C, decreased rapidly at the beginning of fermentation and then reached pH 3.96 and pH 3.62. In both groups, the genera *Lactobacillus*, *Leuconostoc*, and *Weissella* were predominant. Our result suggests that microbial communities during kimchi fermentation may be affected by the fermentation parameters, such as temperature and period, and not enterotoxigenic *E. coli* (ETEC).

## Introduction

Kimchi is a traditional Korean fermented food manufactured using vegetables and condiments [1, 2]. Kimchi has become popular worldwide because of its health-promoting effects, such as antioxidant, antimutagenic, anticarcinogenic, and antihypertensive activities [3]. These properties are derived from various secondary metabolites of lactic acid bacteria (LAB), which are produced during kimchi fermentation.

Kimchi is naturally fermented by various microorganisms present in the raw materials without using a starter culture. The flavor of kimchi depends on the fermentation conditions and LAB included in the fermentation process. Kimchi fermentation leads to the growth of various LAB, such as the genera *Lactobacillus*, *Leuconostoc*, and *Weissella*. The composition of this microbial community is constantly changing throughout the kimchi fermentation process [2, 4]. As LAB increase during fermentation, organic acids, carbon dioxide, mannitol, ethanol, bacteriocins, and vitamins are produced [5, 6]. However, unusual conditions, including early-stage fermentation (when the number of LAB is relatively low) or inadequate fermentation process, may cause food poisoning bacteria to grow. Although rare, food poisoning cases after ingesting fermented foods such as fermented sausage are reported worldwide [7].

Kimchi is generally recognized as a microbiologically safe fermented food despite that some studies have reported *Escherichia coli* can survive in the early stages of fermentation in commercial kimchi due to an inadequate fermentation process [8]. Among the *E. coli* strains, enterotoxigenic *E. coli* (ETEC) causes diarrheal illnesses that affect individuals of different age groups [9]. Furthermore, it has been reported and increasingly recognized as an important cause of foodborne illnesses, such as food poisoning [9]. In general, ETEC doses greater than 10^6^ organisms are an imminent cause of diarrhea in adults [10]. However, the dose response relationship for children is unknown. The probability of infection associated with pathogen intake could alter depending on the actual situation of a population living in an environment with acquired immunity, coinfections, host susceptibility and exposure [11]. Therefore, to estimate the health risk of pathogenic *E. coli*, a quantitative microbial risk assessment (QMRA) is used to evaluate the population exposure in the supply chain using data on the occurrence of pathogenic *E. coli* [12].

Another previous study reported an investigation to identify the source of infection in the ETEC outbreak which occurred in schoolchildren associated with consumption of kimchi through retrospective cohort studies and PFGE (pulse-field gel electrophoresis) pattern analysis [13]. These studies and analysis helped determine the route of transmission and source of infection, but could not identify the microbial community.

To exclude or reduce the risk of contamination of fermented foods and kimchi, it is necessary to investigate the survival of foodborne pathogens and the proliferation of LAB during kimchi fermentation under various parameters, such as fermentation temperature and period [8, 14]. A culture-independent analysis is for analyzing microbial communities directly from a sample [15]. Polymerase chain reaction with denaturing gradient gel electrophoresis (PCR-DGGE) and high-throughput sequencing are commonly used culture-independent methods and widely applied for microbial community analysis. Unlike PCR-DGGE, which is relatively labor-intensive, inaccurate, and time-consuming, high-throughput sequencing has proven to be accurate and rapid for profiling complex microbial communities. Therefore, it has been applied to various environments, including fermented food such as kimchi [2, 16-18], jeotgal [19], and soybean pastes [20, 21]. Despite the importance of evaluating kimchi’s safety, which involves spontaneous fermentation, studies on the stability evaluation of food poisoning bacteria in kimchi are rare [22]. Thus, studies are needed to evaluate the effect or safety of microbial diversity and pathogenic strains in kimchi during fermentation via high-throughput sequencing targeting multiple hypervariable regions.

In this study, high-throughput sequencing was applied to analyze the microbial community of kimchi challenged with selected ETEC MFDS 1009477 strain and fermented at room and low temperature over 21 days. In addition, a more accurate microbial community was identified by targeting seven hypervariable regions of the 16S rRNA gene sequence.

## Materials and Methods 

### Kimchi Preparation and Sampling

Kimchi samples were prepared by mixing salted Napa cabbage and ingredients including radish, garlic, red pepper, and fermented seafood. Only one batch of kimchi was used for this work. An industrial-scale batch of kimchi (100 kg) was divided into a control sample and a sample inoculated with ETEC immediately after production. The control sample was not subjected to any treatment after the kimchi production process. For the preparation of a sample, the inoculated ETEC MFDS 1009477 strain, isolated from radish, was cultured in tryptic soy agar (TSA; BD Diagnostic Systems, USA) at 37°C and incubated for 18 h. The cultured strain was centrifuged at 15,871 ×*g* for 1 min. A pellet was suspended in saline, and the cell suspension was diluted into 5 log colony forming units (CFU/g) of final inoculum into 1 kg of kimchi. After inoculation with the pathogens, the samples were kept at room temperature (25°C) and sales rack temperature (10°C) for 21 days. During fermentation, samples were taken for analyses on the 0.5^th^, 1^st^, 2^nd^, 3^rd^, 5^th^, 7^th^, 9^th^, 11^th^, 14^th^, 17^th^, and 21st day.

### Measurement of pH and Viable Cell Counts

The pH value of kimchi soup was measured using a pH meter (Orion 3-Star Plus pH Meters, Thermo Fisher Scientific, USA). For viable cell counting, the kimchi soup (1 ml) was diluted with 9 ml saline with serial dilution; 1 ml of dilution solution was inoculated into 3M Petrifilm Aerobic Count Plates (3M Petrifilm, USA) before counting total bacterial cells and 3M Petrifilm *E. coli*/Coliform Count plates (3M Petrifilm) for counting *E. coli*. After incubating the Petrifilm at 37°C for 24 h, the number of bacteria and *E. coli* was counted, and the following results were expressed as log CFU/g. Viable cell count was performed on a dilution plate with 15 to 300 colonies. The pH and viable cell counts were carried out in triplicates.

### DNA Extraction and Real-Time PCR

The genomic DNA of the kimchi samples was extracted according to the previous studies [2, 23]. Genomic DNA was extracted by adding 10 ml of kimchi soup to 90 ml of saline and vortexing the mixture. The residual particulates were washed with saline after being centrifuged at 1,000 ×*g* for 5 min, and the supernatants were transferred to new sterile conical tubes. The solution was centrifuged at 4,000 ×*g* for 20 min at 4°C to harvest the bacterial cells. The pellets were resuspended with 5 ml of lysis buffer (20 mM Tris-HCl (pH 8.0), 2 mM EDTA and 1.2% Triton X-100) and lysozyme solution (20 mg/ml) and incubated at 37°C for 30 min. After that, 125 ul of proteinase K (Qiagen, Germany) and 1 ml of AL buffer (Qiagen) were added to the solution and incubated at 56°C for 1 h. The resultant was mixed with 1 ml ethanol (99%), and the supernatant was applied to a DNeasy Blood and Tissue Kit (Qiagen) to extract bacterial genomic DNA. The DNA quality was assessed using a Qubit dsDNA HS Assay Kit (Thermo Fisher Scientific) according to the manufacturer’s instructions.

The real-time PCR for detecting ETEC strain was performed according to the Food Poisoning Cause Investigation Method of the Ministry of Food and Drug Safety [24]. Real-time PCR was performed on a 7500 Fast Real-Time PCR System (Applied Biosystems, USA) to verify ETEC-specific virulence genes. Amplification of the heat-stable enterotoxin gene (*estA*) encoding STp was done by a PowerCheck 15 Pathogen Multiplex Real-Time PCR Kit (Kogene Biotech, Korea). The 20 μl PCR mixture consisted of 5 μl extracted DNA and 15 μl PCR premix. The reactions were carried out under 50°C for 2 min and 95°C for 10 min before the first cycle, followed by 40 amplification cycles of 95°C for 15 s and 60°C for 1 min. A threshold cycle (Ct) value was calculated and compared using bacteria gene detection. A standard curve was constructed using genomic DNA from ETEC MFDS 1009477 strain (10^3^ to 10^8^ CFU/ml) determined by plate counting (Fig. S1).

### Bacterial 16S rRNA Gene Amplification and Sequencing

According to the manufacturer’s protocol, at least 3 ng of total DNA was used for 16S and DNA library preparation. The bacterial population was analyzed by amplifying the 16S rRNA genes using a 16S Metagenomics Kit (Thermo Fisher Scientific) with V2-4-8 and V3-6,7-9 primers, targeting the seven hypervariable regions (V2, V3, V4, V6-7, V8, and V9) of bacterial 16S rRNA genes. Amplicon lengths of V2, V3, V4, V6-7, V8, and V9 hypervariable regions were 250, 215, 288, 260, 295, and 209 bp, respectively. The targeted 16S sequencing libraries were amplified using the Ion Plus Fragment Library Kit and Ion Xpress Barcode Adapters Kit (Thermo Fisher Scientific). The amplified DNA was purified using Agencourt AMpure XP beads (Beckman Coulter, USA) on DynaMag-2 Magnet (Thermo Fisher Scientific). The concentration of purified library was measured on an Agilent 2100 Bioanalyzer using an Agilent High Sensitivity DNA Kit (Agilent Technologies, USA). Then, the library was diluted into a final concentration of 100 pM. The Ion Chef instrument was used to perform clonal amplification of the pooled library on ion sphere particles by emulsion PCR, bead enrichment, and chip loading using the Ion Torrent 510 & 520 & 530 Kit Chef Template Preparation System (Thermo Fisher Scientific). Load ion 530 chips were sequenced using the Ion Torrent S5 System (Thermo Fisher Scientific) with 850 flows.

### Data Processing

After sequencing, the reads of low quality, low abundance (< 10 reads), reads less than 150 bp, and polyclonal reads were filtered and uploaded to the Ion Reporter server. Then they were analyzed based on the customized Metagenomic 16S w1.1 workflow, consisting of the curated MicroSEQ ID and SILVA version 138 as reference database using Ion Reporter software version 5.16 [25]. Throughout the analysis of OTUs (operational taxonomic units), alpha and beta diversity was performed using Quantitative Insights into Microbial Ecology (QIIME). The reads were assigned to the database at the 97% identity cut-off for genus level to classify the OTU-based analysis. The alpha diversity analysis, which includes Chao1, Shannon and Simpson (Gini-Simpson) indexes, was used to analyze their abundance within the samples. The beta diversity was analyzed among microbial communities in samples using two-dimensional Principal Coordinates Analysis (PCoA) based on the Bray–Curtis dissimilarity. The PCoA plots were used to visualize the microbiota distances among the sample groups.

### Statistical Analysis

Statistical analysis of pH value was performed using R software (ver. 4.1.0) to identify significant differences (*p* < 0.05) using Duncan’s multi-rage test. Significant differences between groups of microbial communities were calculated using the Mann–Whitney U test in R software (ver. 4.1.0). Principal Coordinates Analysis (PCoA) was tested using analysis of similarities (ANOSIM) as implemented in the vegan R package. Results with *p* < 0.05 were considered significant.

## Results and Discussion 

### Changes in pH

**Fig. 1:**
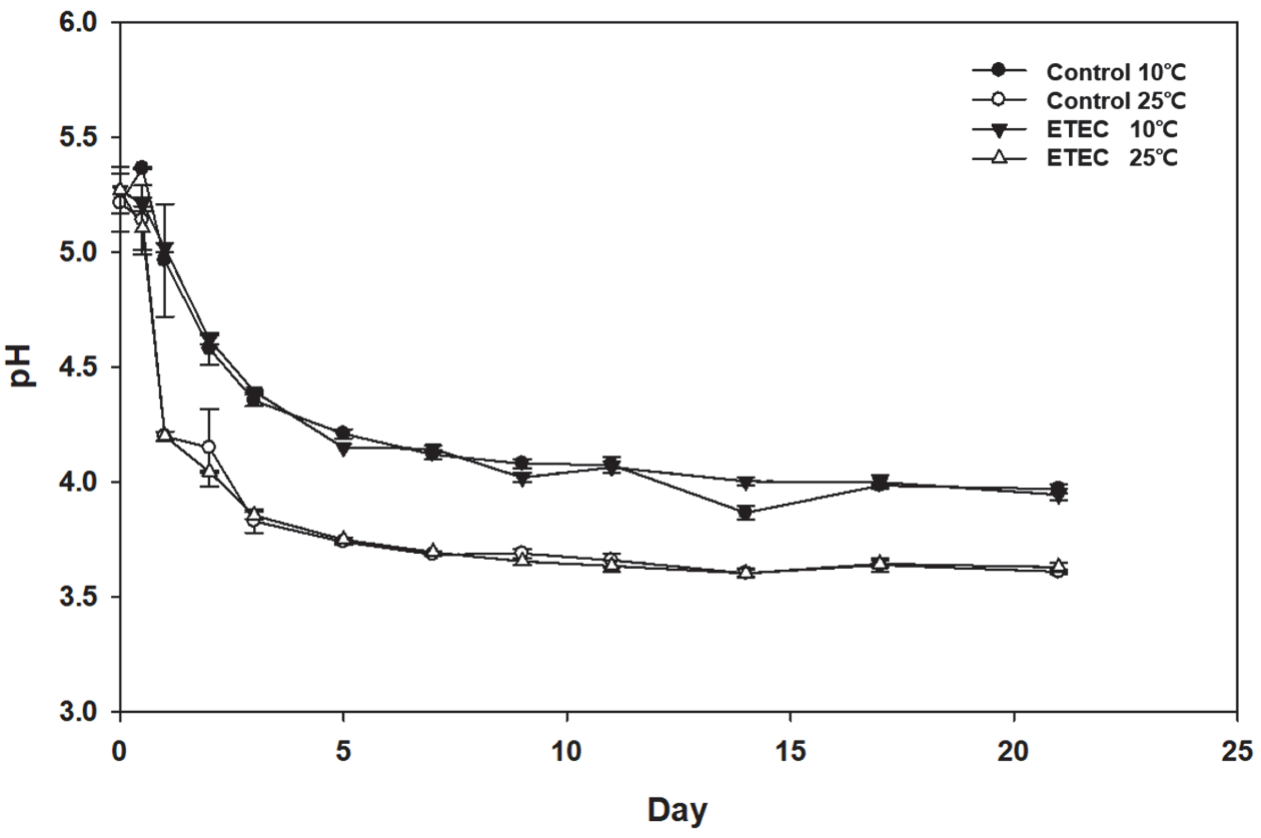
Change of pH in kimchi during fermentation at 10 and 25°C. The kimchi samples are as follows: ETEC, 10^5^ CFU/g enterotoxigenic *Escherichia coli* spiked into kimchi; control; negative control. Error bars represent the standard deviations among the three replicates.

The pH values in the fermented kimchi were monitored every sampling time. The pH value immediately after preparation of the kimchi sample was 5.24. Overall, the pH value decreased gradually over fermentation time and decreased more slowly at the lower temperature condition (10°C) than at room temperature (25°C) (Fig. 1 and Tables S1). At each fermentation temperature, the decrease in pH value was similar between the two groups (control and ETEC). In general, a pH value of 4.2 is considered optimal for samples of the highest quality kimchi [17]. At 10°C, both groups took about five days to reach a pH of 4.2, while it took about one day at 25°C. Both groups decreased rapidly up to pH 4.2; then, the pH value remained stable until the end of fermentation (21 days).

This means that the fermentation temperatures affected the pH decrease. This result is consistent with a previous study that LAB strains that produce organic acids (lactic acid and acetic acid) during fermentation grow much faster at room temperature than at low temperature [26]. Moreover, the LAB strains entered the log growth phase more rapidly at room temperature (25°C) than at low temperatures. The pH value decreased rapidly due to the production of many organic acids while remaining relatively stable during late fermentation [27].

### Abundance and Diversity of Bacterial Communities

High-throughput sequencing of the 16S rRNA gene fragment was performed for 46 fermented kimchi samples. As shown in Supplementary Tables S2-S3, the 0.5 day sample showed a remarkable difference in Chao1 richness estimators compared with the 0 day sample. The Chao1 index of the 0.5 day sample in the 10 and 25°C fermented kimchi was higher than that of other samples. Comparing the Chao1 index according to the kimchi sample inoculated with the pathogens showed that the overall pattern was similar to the kimchi sample inoculated without the pathogen. In the Shannon and Simpson indices, decreases in the diversity indices changed more slowly at the lower temperature (10°C) than at the higher temperature (25°C). The change in diversity indices of the kimchi sample inoculated with the pathogen was similar to that of the kimchi sample inoculated without the pathogens.

This means that the bacterial community in kimchi differed according to fermentation temperature and period. Particularly, the 0.5 day sample in the 10- and 25°C-fermented kimchi showed the highest OTU richness and diversity. Additionally, the reducing rate was different depending on the fermentation temperature.

### Comparison of Microbial Community among Kimchi Samples

The amplification of 16S rRNA hypervariable regions can be used to detect microbial communities in a sample typically down to the genus level. For each individual sample, amplicons were sequenced for a total of 1,464,725 reads, of which 1,379,320 were mapped.

**Fig. 2:**
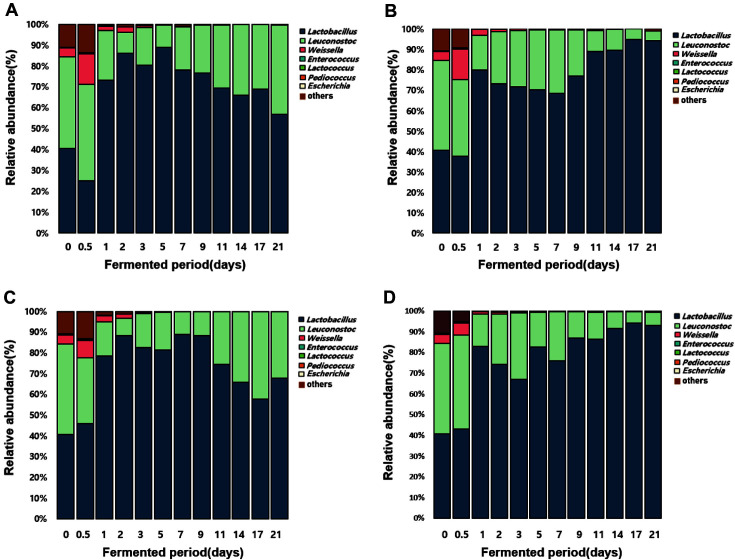
Composition of microbial communities at the genus level in kimchi fermented at (**A**) 10°C and (**B**) 25°C. Composition of microbial communities at the genus level in kimchi inoculated with ETEC fermented at (**C**) 10°C and (**D**) 25°C.

The taxonomic classifications of sequences for the two groups at each fermentation temperature and period are represented in Fig. 2. In the kimchi immediately after preparation, *Lactobacillus*-related genera (38.23%), *Leuconostoc* (41.04%), and *Weissella* (4.13%) exhibited the highest abundance of LAB. The relative proportion showed that genera (83.40%) involved primarily in kimchi fermentation, such as *Lactobacillus*-related genera, *Weissella*, and *Leuconostoc*, were dominant in the microbial communities in all fermented kimchi samples. The result is consistent with those of previous studies, which showed that *Lactobacillus*-related genera, *Weissella*, and *Leuconostoc* were the most dominant ones during kimchi fermentation [17]. *Lactobacillus*-related genera, *Weissella*, and *Leuconostoc* were present during 21 days of the kimchi fermentation at 10 and 25°C, and *Weissella* decreased gradually; meanwhile, *Lactobacillus*-related genera were dominant until the end of fermentation. The change of the microbial communities in the ETEC group, the sample inoculated with the ETEC MFDS1009477 strain, was similar to that of the control group, the sample inoculated without the ETEC strain. In the control group, genus *Escherichia* was not detected at either of the fermentation temperatures. At 10°C, the ETEC group was detected in the genus *Escherichia* (0.2%) until day 1 of fermentation but was not detected thereafter. At 25°C, the genus *Escherichia* (0.4%) was not detectable after 0.5 days. Comparison of relative abundances showed that *Escherichia* genera were very low and there were no significant differences between control and ETEC group due to the relative dominance of LAB in all fermented kimchi samples.

According to a previous survival study of *E. coli*, the contamination of kimchi with *E. coli* O157:H7 remained at a high level throughout the incubation period. The survival studies involved determining numbers of pathogenic bacteria that could be recovered from inoculated kimchi. The initial counts of *E. coli* O157:H7 for kimchi were 5.22 to 5.30 log CFU/g; however, after 7days of incubation, *E. coli* showed a decreased of 1.0. to 2.0 log CFU/g. Pathogenic bacteria like *E. coli* O157:H7 were able to survive under acidic conditions (pH ≥ 4.0) for up to 54 days but were affected by acidulants and temperature [28]. Other serotypes of *E. coli* have been shown to survive for at least 2 days in traditional lactic acid-fermented foods [29, 30].

Additionally, survival studies of pathogenic bacteria during kimchi fermentation were performed by inoculating kimchi with pathogenic bacteria at a concentration of 5.0 ~ 7.0 log CFU/g [8, 14, 31]. Through these studies, it was confirmed that there was no significant difference in the survival rate of pathogens according to the inoculation concentration during kimchi fermentation.

In this study, we also spiked into kimchi samples 5 log CFU/g of ETEC and confirmed that the number of viable *E. coli*, including ETEC strains, decreased more rapidly in the 25°C sample than in the 10°C sample and they were no longer detected after two and seven days, respectively (Fig. S2). Since all *E. coli* strains, including ETEC, can be counted on the *E. coli*/coliform count plate, real-time PCR was performed to detect ETEC strains specifically. The *estA* gene was detectable until days 14 and 7 in kimchi fermented at 10 and 25°C, respectively (Fig. S3). The ETEC might have survived late fermentation, but DNA derived from dead cells may have been detected in a PCR reaction. These results showed that ETEC does not affect the formation and change of microbial community during the kimchi fermentation process.

The pH change is caused by organic acids produced during fermentation, and the resulting low pH of fermented food is an important factor that inhibits the growth of pathogenic bacteria [8]. In particular, previous studies have reported that high acidity and low pH in food were related to the decrease of *E. coli* [32]. *Lactobacillus*-related genera, *Leuconostoc*, and *Weissella*, which play an important role in kimchi fermentation, are bacteria that produce organic acids, bacteriocin, and flavor ingredients during kimchi fermentation [11, 27, 33]. Also, these genera produce organic acids (lactic acid and acetic acid) from carbohydrates during kimchi fermentation, lowering the pH value according to the fermentation stage and causing changes in the microbial communities [27, 34]. In this study, ETEC was decreased with an increase in *Lactobacillus*-related genera, which may be associated with bacteriocin or organic acid production. These present study results suggest that the antimicrobial substances or pH changes caused by the dominant genera in kimchi contribute to the decrease of ETEC populations during fermentation. However, since this study did not confirm the possibility of bacteriocin production of *Lactobacillus*-related genera in kimchi, further studies are needed to discover which factors inhibit pathogenic bacterial strains during fermentation.

**Fig. 3:**
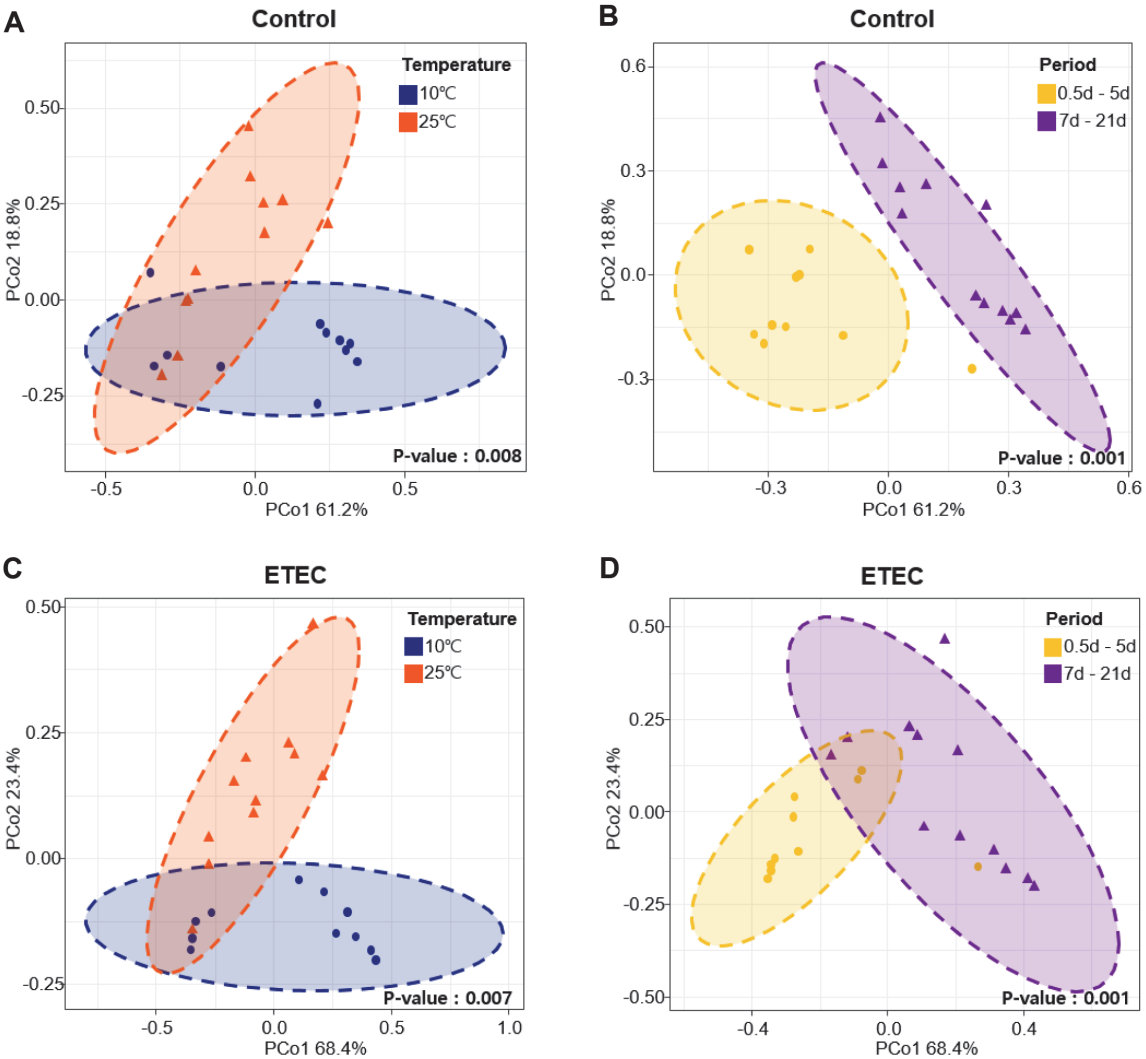
Principal Coordinates Analysis (PCoA) plot based on Bray-Curtis dissimilarity according to fermentation period and temperature. (**A-B**) control; (**C-D**) ETEC.

**Fig. 4:**
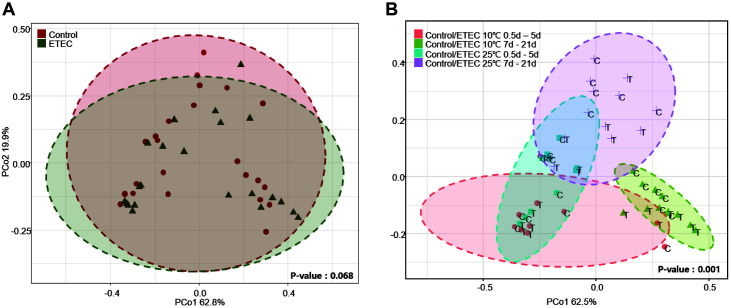
Principal Coordinates Analysis (PCoA) plot based on Bray-Curtis dissimilarity between control and ETEC. (**A**) control and ETEC; (**B**) control and ETEC according to fermentation period and temperature.

Beta diversity was illustrated using PCoA and showed the similarities in the bacteria composition across different samples. Bacteria composition changes in kimchi samples inoculated with or without the pathogens were analyzed in PCoA plots based on the Bray–Curtis dissimilarity. The variation among bacterial composition based on fermentation period and temperature in the kimchi sample inoculated without the pathogens was statistically significant (ANOSIM, *p* < 0.05; Figs. 3A and 3B). In addition, the variation among bacterial composition based on fermentation period and temperature in the kimchi sample inoculated with ETEC was statistically significant (ANOSIM, *p* < 0.05; Figs. 3C and 3D). As a result, the bacteria in the kimchi samples inoculated with or without the pathogens represented four clusters according to the fermentation period (0~5 days, 7~21 days) and temperature (10°C, 25°C) (ANOSIM, *p* = 0.001; Fig. 4). These findings revealed that the bacterial communities of the kimchi samples showed a close relationship between fermentation period and temperature. Particularly, our data suggest that pathogenic *E. coli* does not affect the formation and change of microbial community.

In this study, we confirmed the effect of ETEC during kimchi fermentation at low temperature (10°C) and room temperature (25°C) using high-throughput sequencing targeting seven hypervariable regions. We found that the reduction of pH may cause the inhibitory effect of pathogenic *E. coli* in kimchi according to the growth of LAB. In particular, the pH was decreased rapidly at room temperature (25°C) by LAB, which proliferated better at room temperature than at low fermentation temperature (10°C). The difference in the microbial communities was not observed between the control and ETEC groups, and the genus *Escherichia* community was not detectable at both temperatures after one day in the ETEC group. Our results suggest that fermentation parameters, such as temperature and period, can naturally control unintentionally inoculated ETEC. In further studies, we may need to research various serotypes and pathotypes of *E. coli* during the kimchi fermentation process.

## Supplemental Materials

Supplementary data for this paper are available on-line only at http://jmb.or.kr.
