# Supplementary material for: Effect of Enterotoxigenic Escherichia coli on Microbial Communities during Kimchi Fermentation
Source: J Microbiol Biotechnol. 2021 Sep 8;31(11):1552–8. doi: 10.4014/jmb.2108.08038 (PMC9705866; doi:10.4014/jmb.2108.08038)
Supplement: Supplementary file 1 [file jmb-31-11-1552-supple.pdf]

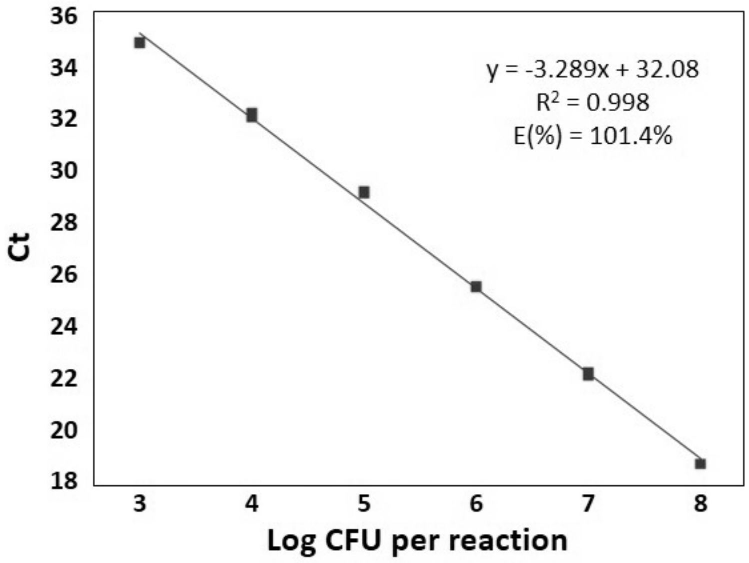

Fig. S1. A standard curve for specific primer targeting *estA* gene.

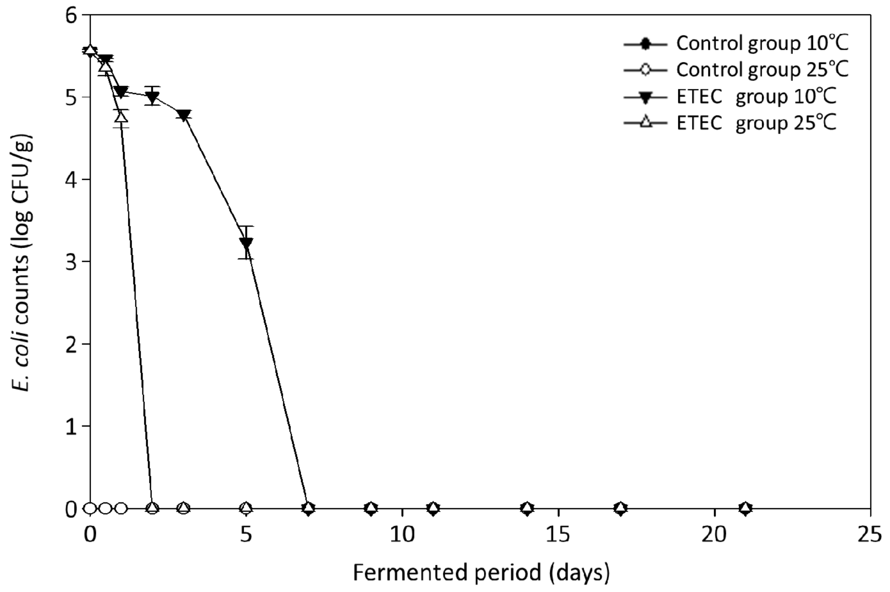

Fig. S2. Number of *E. coli* cells in kimchi during fermentation at 10 and 25℃. The kimchi samples are as follows: ETEC, 10^5 CFU/g enterotoxigenic *Escherichia coli* spiked into kimchi; control; negative control. Error bars represent the standard deviations among the three replicates.

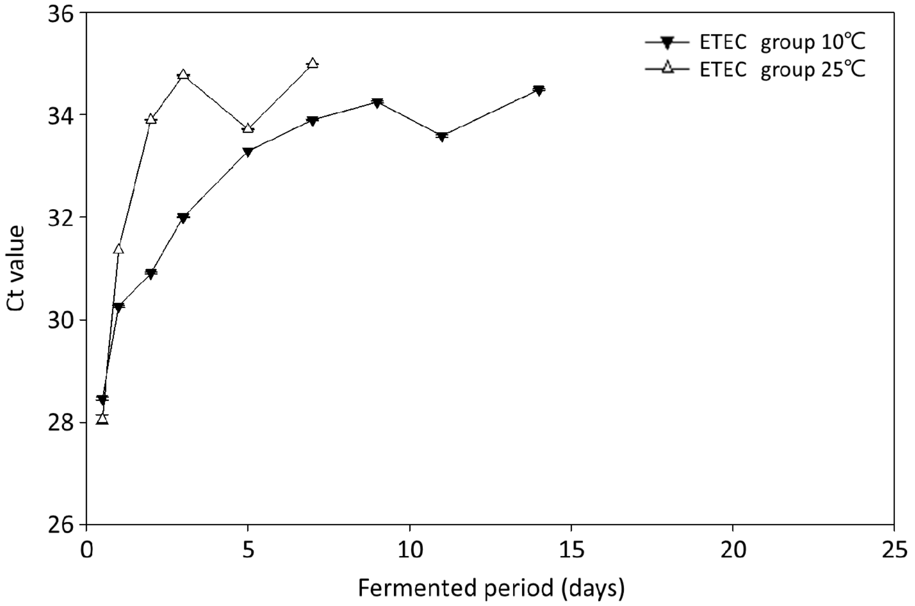

Fig. S3. Ct values of *estA* gene in kimchi during fermentation at 10 and 25℃. The kimchi samples are as follows: ETEC, 10^5 CFU/g enterotoxigenic *Escherichia coli* MFDS 1009477 spiked into kimchi. Error bars represent the standard deviations among the two replicates.

Table S1. Difference in pH value according to the fermentation temperatures.

Fermented period (days)	Control group		ETEC group	
	10°C	25°C	10°C	25°C
0	5.22±0.13[a]	5.22±0.13[a]	5.27±0.10[a]	5.27±0.10[a]
0.5	5.37±0.00[a]	5.14±0.15[a]	5.21±0.03[a]	5.11±0.10[a]
1	4.97±0.25[a]	4.20±0.02[b]	5.02±0.02[a]	4.20±0.00[b]
2	4.58±0.07[a]	4.27±0.24[a]	4.62±0.02[a]	4.05±0.00[b]
3	4.36±0.02[a]	3.83±0.05[b]	4.40±0.01[a]	3.86±0.02[b]
5	4.21±0.02[a]	3.74±0.01[b]	4.15±0.00[a]	3.75±0.01[b]
7	4.12±0.02[a]	3.69±0.00[b]	4.15±0.02[a]	3.70±0.01[b]
9	4.08±0.02[a]	3.69±0.02[b]	4.02±0.02[a]	3.66±0.01[b]
11	4.08±0.04[a]	3.66±0.03[b]	4.07±0.02[a]	3.64±0.03[b]
14	3.80±0.01[a]	3.37±0.01[b]	4.00±0.02[a]	3.60±0.02[b]
17	3.99±0.01[a]	3.64±0.03[b]	4.00±0.03[a]	3.65±0.02[b]
21	3.97±0.02[a]	3.61±0.01[b]	3.95±0.03[a]	3.63±0.02[b]

Data values indicated as the mean ± SD of three replications. Values followed by different letters (a-b) are significantly different ($p<0.05$).

Table S2. Composition of Chao1, Shannon, and Simpson index in samples of control group.

Fermented period (days)	Richness estimators		Diversity indices			
	Chao1		Shannon		Simpson	
	10°C	25°C	10°C	25°C	10°C	25°C
0	88		1.69		0.56	
0.5	225	186	2.54	2.28	0.70	0.70
1	37	27	1.03	0.87	0.41	0.33
2	54	24	0.77	0.94	0.25	0.40
3	17	12	0.81	0.93	0.32	0.41
5	14	12	0.52	0.92	0.20	0.42
7	15	12	0.86	0.96	0.35	0.44
9	12	16	0.81	0.82	0.36	0.36
11	13	10	0.91	0.56	0.43	0.20
14	16	7	0.94	0.5.	0.45	0.19
17	9	5	0.91	0.30	0.43	0.10
21	13	24	1.01	0.40	0.49	0.11

Table S3. Composition of Chao1, Shannon, and Simpson index in samples of ETEC group.

Fermented period (days)	Richness estimators		Diversity indices			
	Chao1		Shannon		Simpson	
	10°C	25°C	10°C	25°C	10°C	25°C
0	83		2.06		0.64	
0.5	140	80	2.38	1.77	0.68	0.61
1	101	21	1.06	0.75	0.36	0.29
2	39	14	0.68	0.92	0.21	0.39
3	19	6	0.73	0.99	0.29	0.45
5	22	18	0.73	0.72	0.30	0.29
7	13	19	0.51	0.83	0.20	0.37
9	12	13	0.53	0.59	0.21	0.23
11	13	15	0.84	0.62	0.38	0.24
14	18	12	0.94	0.44	0.45	0.16
17	27	15	1.01	0.35	0.49	0.11
21	15	14	0.93	0.41	0.44	0.13
